# Nanoparticles as a Tool for Alleviating Plant Stress: Mechanisms, Implications, and Challenges

**DOI:** 10.3390/plants13111528

**Published:** 2024-05-31

**Authors:** Ankita Kumari, Ashish Kumar Gupta, Shivika Sharma, Vikash S. Jadon, Vikas Sharma, Se Chul Chun, Iyyakkannu Sivanesan

**Affiliations:** 1 Molecular Biology and Genetic Engineering Domain, School of Bioengineering and Bioscience, Lovely Professional University, Phagwara-Jalandhar 144411, Punjab, India; anki2026as@gmail.com (A.K.); shivikasharma25@gmail.com (S.S.); biotech_vikas@rediffmail.com (V.S.); 2 ICAR—National Institute for Plant Biotechnology, Pusa Campus, New Delhi 110012, India; ashish.pathology@gmail.com; 3 School of Biosciences, Swami Rama Himalayan University, JollyGrant, Dehradun 248016, Uttarakhand, India; vsjadon@srhu.edu.in; 4 Department of Environmental Health Science, Institute of Natural Science and Agriculture, Konkuk University, Seoul 05029, Republic of Korea; scchun@konkuk.ac.kr

**Keywords:** oxidative stress, antioxidants, nanoparticles, reactive oxygen species

## Abstract

Plants, being sessile, are continuously exposed to varietal environmental stressors, which consequently induce various bio-physiological changes in plants that hinder their growth and development. Oxidative stress is one of the undesirable consequences in plants triggered due to imbalance in their antioxidant defense system. Biochemical studies suggest that nanoparticles are known to affect the antioxidant system, photosynthesis, and DNA expression in plants. In addition, they are known to boost the capacity of antioxidant systems, thereby contributing to the tolerance of plants to oxidative stress. This review study attempts to present the overview of the role of nanoparticles in plant growth and development, especially emphasizing their role as antioxidants. Furthermore, the review delves into the intricate connections between nanoparticles and plant signaling pathways, highlighting their influence on gene expression and stress-responsive mechanisms. Finally, the implications of nanoparticle-assisted antioxidant strategies in sustainable agriculture, considering their potential to enhance crop yield, stress tolerance, and overall plant resilience, are discussed.

## 1. Introduction

**Figure 1:**
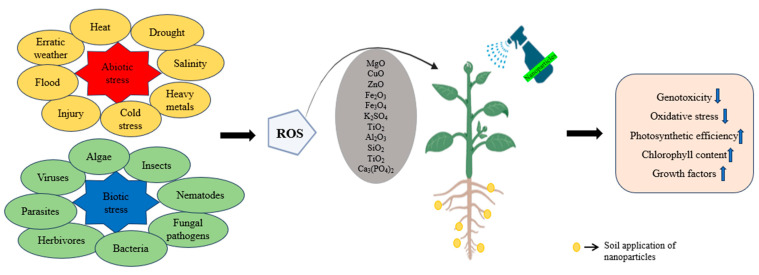
Generalized overview of plant in response to stress and its mitigation by application of different nanoparticles (foliar and soil application).

Global biomass production from agricultural farmlands is challenged by varietal environmental stresses [1,2,3]. Being sessile, plants are constantly exposed to these environmental stressors, which are generally categorized as biotic and abiotic stressors [4,5,6,7,8]. The main biotic stressors include pathogens, insects, and herbivores, while abiotic stressors include heavy metal exposure, soil salinity, erratic weather patterns, and climate change [7,9]. Consequently, these stresses induce a cascade of bio-physiological changes in plants, ultimately affecting their overall health and productivity [6,10]. One prominent consequence of these stressors is oxidative stress, which is identified by an imbalance in the antioxidant defense system [7,11]. Reactive oxygen species (ROS) are produced as a natural byproduct of standard metabolic pathways involving oxygen [12,13]. Principally, the sites of ROS generation include apoplast, chloroplast, mitochondria, and peroxisomes [14,15]. These ROS can potentially lead to DNA damage (by affecting nucleic acids), enzyme inhibition (due to oxidation of proteins), and lipid peroxidation, eventually inducing cell injury, bursting cell organelles, and causing programmed cell death [16] (Figure 1). As a coping mechanism, plants have evolved various intricate mechanisms against diverse environmental stressors [17]. Normally, there is a balance in the production and elimination of ROS within the cell. However, external stressors hamper the production–elimination balance resulting in the excess generation and accumulation of ROS [18,19]. Consequently, rapid leakage of ROS occurs, which further alters the metabolic, morphological, and physiological processes of the plant [20,21,22]. To counteract the deleterious effects of ROS, plants have evolved complex enzymatic and non-enzymatic defense mechanisms collectively called the “antioxidant system” [23,24]. The enzymes of antioxidant system include ascorbate peroxidase (APX), Catalase (CAT), dehydro-ascorbate reductase (DHAR), glutathione reductase (GR), glutathione peroxidase (GPX), glutathione S-transferase (GST), mono-hydro ascorbate reductase (MDAR), peroxide reduction (PRX) and superoxide dismutase (SOD). The non-enzymatic antioxidants include ascorbic acid (AA), α-tocopherol, carotenoids, flavonoids, glutathione (GSH), and plastoquinone/ubiquinone [25,26,27]. Both groups of antioxidants are necessary for ROS homeostasis, and previous studies suggest that high antioxidative activity is linked to stress tolerance in plants and thus plays a pivotal role in adaptation to stress in plants [28,29,30].

In recent years, the advancement in nanotechnology has been observed to be aligned with the study of nanoparticles in plants, as it can trigger the various enzymatic and non-enzymatic antioxidant capabilities of plants. Nanoparticles are natural or artificially synthesized particles, having sizes ranging from 1 to 100 nanometers. As compared to their bulk materials, nanoparticles have different properties; however, their effects vary according to their concentrations [31,32]. Previous studies suggest that higher concentrations (up to 2000 mgL^−1^) in the application of nanoparticles negatively affects the biochemistry, morphology, and physiology of plants, as well as causing genotoxicity [33,34,35], while application at appropriately standardized concentrations causes positive effects [36,37,38,39]. The current study presents a comprehensive review of the role of nanoparticles in stress amelioration through redox homeostasis and by improving the antioxidative system in plants. Furthermore, the use of different nanoparticles and their role in mediating biochemical, physiological, proteomic, and gene expression changes are discussed.

## 2. Environmental Stressors and Their Impact on Plants

### 2.1. Abiotic Stressors

**Figure 2:**
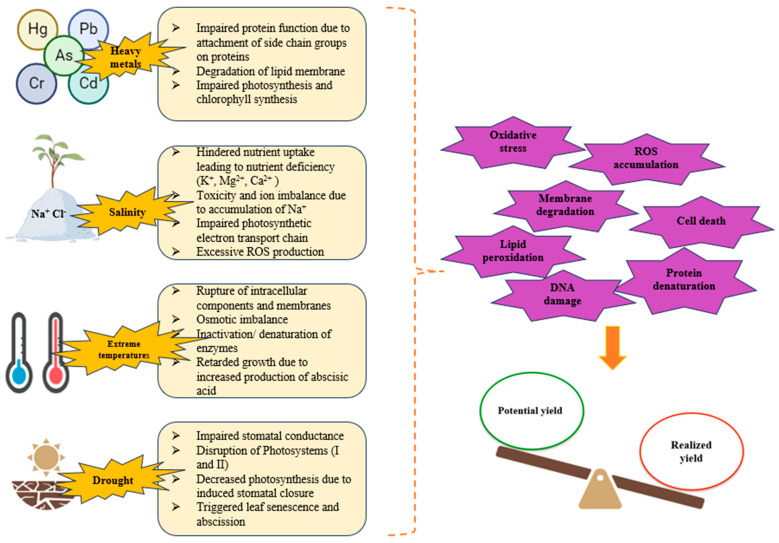
Varietal abiotic stressors affecting plant metabolism, physiology and morphology resulting in poor growth and productivity of plants (lower yield).

The term abiotic stressors refers to all the nonliving entities which negatively impact the metabolism and growth of plants. Heavy metal accumulation in soil, drought, salinity, erratic weather conditions, and extreme low and high temperatures, all contribute to abiotic stress in plants that greatly affect agriculture worldwide, consequently, leading to massive economic losses. In addition to natural causes such as climate change and global warming, various anthropogenic activities, such as intensive agriculture, rapid industrialization and rising population, indirectly trigger abiotic stress (Figure 2). For instance, drought and salinity can hinder water uptake, impairing plant physiological processes and reducing crop yields; extreme temperatures can cause thermal stress, which damages cellular structures and inhibits enzyme activity; heavy metal accumulation, such as cadmium, lead, and arsenic, in the soil can lead to phytotoxicity, disrupting cellular processes by generating reactive oxygen species (ROS) that damage DNA, proteins, and lipids. This metal-induced oxidative stress interferes with photosynthesis and respiration, ultimately stunting plant growth and development. Erratic weather conditions, including unseasonal rain or prolonged dry spells, can disrupt the phenological stages of plants, such as flowering and fruiting, thereby affecting reproductive success and crop yield. Additionally, flooding can lead to hypoxic conditions in the root zone, inhibiting root respiration and nutrient uptake [1,2,3,40,41]. To counteract and promote tolerance, plants activate early stress signaling mechanisms [7]. These include the release of secondary messengers, such as nitric oxide, reactive oxygen species, and calcium, that transmit and amplify the signals as well as activating protein kinases, like SnRk1, which changes the expression of key stress-responsive genes to restore homeostasis in plant cells [42,43,44]. These intricate mechanisms consequently activate the transcription factors that eventually activate various stress responsive genes, thereby facilitating stress tolerance. Besides, releases of phytohormones, including ethylene and Abscisic acid, trigger the activation of stress response.

Under drought stress, major phytohormones, including auxins (AUX), gibberellic acids (GA), cytokinin (CK), and abscisic acid (ABA), have been reported to be decisive in plant adaptation to drought stress. For instance, the ABA signaling genes *OsABI5*, *Oshox22*, *OsNAC5*, *DSM2* in rice have been reported to improve yield in drought stress through ABA biosynthesis. Similarly, induced expression of a CK biosynthetic gene, isopentyl transferase (IPT), is known to increase CK levels, thereby protecting the plant by delaying drought-induced senescence. Another gene *DRO1*, upon higher expressions and improved drought tolerance, is negatively regulated by Auxin. In addition, decreased levels of GA aligned with decreased plant growth [45]. Accumulation of late embryogenesis-abundant (LEA) mRNA was also observed upon downregulation of IAA [46]. A similar study on barley observed a fivefold increase of ABA in drought tolerant varieties as compared to susceptible ones [47]. Overexpression of ABA biosynthesis gene *NCED3* (9-cis-epoxycarotenoid dioxygenase) in *Arabidopsis* resulted in improved shoot growth under drought stress [48]. In maize, ABA deficiency resulted in increased ethylene production and triggered ethylene-induced leave senescence [49].

Studies suggest that transcription factors are critical in mediating abiotic stress tolerance upon overexpression [50]. Aligning with this, various transcription factors have been reported for promoting abiotic stress tolerance, including OsERF1 in rice, GmERF3 in soybean, and *ERF1* in *Arabidopsis* [51,52,53,54]. In transgenic *Arabidopsis*, the transcription factor *SCDREB5* from screw moss regulated jasmonic acid biosynthesis, thereby promoting salinity stress [55]. The upregulation of *OsDREB1A* in *Arabidopsis* has also been linked with salinity tolerance [56]. Moreover, in rice, the upregulation of TF *OsSTAP1* and *OsDREB1B* enhanced salinity tolerance, and the upregulation of *OsDREB2A* and *OsDREB2B* improved salt tolerance in both *Arabidopsis* and rice [57]. Change in temperature is also linked with triggering stress in plants; cold temperature results in inactivation of enzymes, halting cellular machinery and heat results in denaturation of proteins and enzymes [57,58]. In cold stress, a cascade of transcription factors is activated which activates COR genes (cold responsive genes) that regulate the membrane fluidity and inward flow of calcium, e.g., a loss of function mutation at AtANN1 results in promoting freezing tolerance in *Arabidopsis* [59]. Likewise, in heat stress, various heat shock proteins (HSP) are activated that prevent protein denaturation [60]. For instance, in rice and *Arabidopsis*, heat stress (40 °C), HSP70 was activated in a short span of time [61]. Various nanoparticles have been deployed to promote stress tolerance in plants. The use of silicon nanoparticles in different concentrations via two different methods (foliar and soil application) suggested improved plant growth by foliar application, as it contributed to an increased content of photosynthetic pigments and antioxidant enzymes in *Lilium*. Similar results were concluded for silicon nanoparticles in potato plants in drought stress, in wheat plants against *Rhizoctonia solani* infection and in blueberry plants against hypoxia-induced oxidative damage. Similarly, foliar supplementation of rice plants at 90 ppm of silicon dioxide nanoparticles showed improved growth under water regime conditions. Consistent with these findings, application of biosynthesized copper nanoparticles on seeds of *Lens culnaris* revealed that roots treated with 0.025 mgmL^−1^ of copper oxide nanoparticles had the highest activity of enzymes related to the defense system, along with increased total phenolic content. Supplementation of media with zinc oxide nanoparticles also resulted in olive plants in increased chlorophyll a and b content. Likewise, supplementation of culture media with silver nanoparticles suggested that higher concentrations (more than 110 mgdm^−3^) cause decreased activity of antioxidant enzymes (peroxidase, catalase, super oxidase dismutase, ascorbate peroxidase) in lavender. The spray application of boron nanoparticles at 12.5 ppm significantly increased the antioxidant activity of pea plants in drought stress [62,63,64,65,66,67,68,69,70,71,72]. The application of silica nanoparticles in rice for enhanced growth in water regime conditions has been reported [68].

Moreover, a positive effect on plant growth was observed by application of magnetite nanoparticles [73]. Consistent with this, a positive effect on leaf area and shoot length was observed by application of silica nanoparticles in drought stress [74]. In addition, improved photosynthesis and antioxidants in wheat plant were observed by application of iron oxide nanoparticles [75]. Similar studies on banana have been conducted suggesting improved resistance to cold stress by application of chitosan nanoparticles [76].

### 2.2. Biotic Stressors

**Figure 3:**
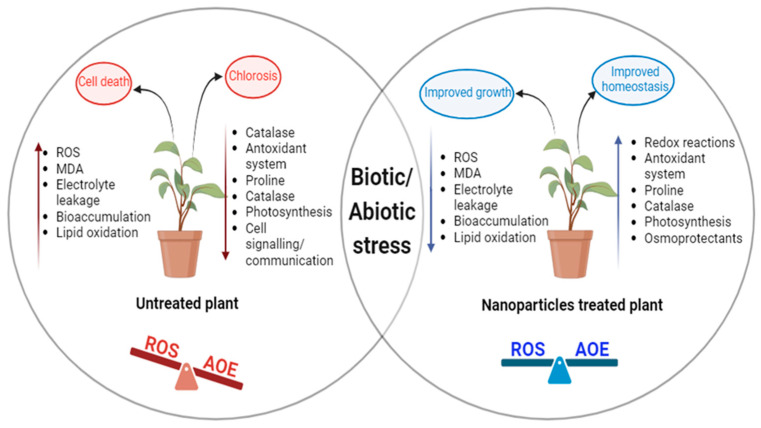
Image describing improved growth of plant upon treatment with nanoparticles in oxidative stress leading to ROS–AOE homeostasis (ROS- Reactive oxygen species; AOE- Antioxidant enzymes).

In agriculture, biotic stress is a major contributor to pre- and post-harvest crop losses [77]. Biotic stress is induced by living entities, more specifically by fungi, viruses, bacteria, insect pests, and herbivores, which unlike abiotic stress drastically hamper plant growth by nutritional deprivation, which potentially causes plant death [78,79] (Figure 1 and Figure 3). With the course of evolution, plants have evolved sophisticated strategies that lead to activation of their defense systems, just as in the case of abiotic stresses. Jasmonic acid (JA) signaling has been reported to be critical in promoting biotic stress tolerance as it induces the production of protease inhibitors, phytoalexins, and key genes required in plant defense [80,81]. In rice, JA-responsive genes *ch11* and *AP24* were observed to induce tolerance to sheath blight [82]. Similarly, the JA-responsive *WRKY* gene in maize has been reported in defense against herbivore attack [83]. Likewise, the *ORA12* gene in *Arabidopsis thaliana* has been reported to be involved in plant defense against diverse biotic stressors [84]. The role of zinc oxide nanoparticles in tomato plants has also been suggested to boost immunity [85].

Accumulation of ROS as response to abiotic and biotic stresses can impair various essential physiological processes of plants. In addition, long term exposure of these stressors might permanently damage plants, thereby affecting the overall yield and productivity of the plants. For mitigating impacts caused by abiotic and biotic stresses on plants, nanoparticles are suggested to be promising. In various studies, nanoparticles have been reported to mitigate varietal abiotic and biotic stresses. The use of various nanoparticles and their roles in alleviating varietal abiotic and biotic stresses have been highlighted in Table 1 and Table 2 in the upcoming sections.

## 3. Nanoparticles and Antioxidant System

### 3.1. Oxidative Stress and Plant Physiology

The production of ROS is a normal part of photosynthesis [86]. However, rapid ROS synthesis leads to its accumulation and activation of the antioxidative system, as discussed in the previous sections [87]. The main consequence of excessive ROS is its oxidative effects on proteins, nucleic acids, lipids, and other cellular organelles leading to cell death [88]. In plants, the chlorophyll content and carotenoids determine photosynthesis rate. These pigments absorb sunlight, and carotenoid helps to provide photoprotection to plants via non-photochemical quenching [89]. The biotic/abiotic oxidative stress induced triggers oxidative damage and ROS leads to destruction of photosynthetic machinery [20]. Preservation of chlorophyll and carotenoids in plants is suggested against numerous stressors, so that plants can continue to perform photosynthesis [90,91]. A few studies have demonstrated the modulation of antioxidant systems in order to understand the physiological, biochemical, and morphological changes in citrus plants upon aging at three different stages, viz. young leaves, mature and senescent leaves. The study observed a gradual decrease in the effect of the non-enzymatic antioxidant system [92]. Similarly, induction of phenolic compounds and expression of ROS detoxification genes was observed to be associated with chitosan in grapevine [93]. Furthermore, in *Arabidopsis*, the effect of H_2_O_2_ on chloro-plastic DJ-1B revealed that H_2_O_2_ decreased glyoxalase activity [94].

Various nano-assisted approaches have been used to improve tolerance to oxidative stress in plants (Table 1 and Table 2). Considering the importance of preservation of chlorophyll and other pigments for photosynthesis in plants, various attempts have been made towards nano-mediated improvement in plant pigments. A recent study on citrus (mandarin oranges) suggested considerable improvement in chlorophyll and carotenoid concentrations in HLB-infected (Huanglongbing) plants by foliar treatments with green synthesized AgNPs (silver nanoparticles). The same study further suggested that the varied amounts of AgNPs enhanced the performance of enzymatic and non-enzymatic antioxidants, including superoxide dismutase, peroxidase, catalase, total phenolics, and flavonoid content. Consistent with the findings, the authors suggested the use of AgNPs at 75 mgL^−1^ as ideal for increasing antioxidant enzymes [95]. In another similar study in rice plants, the application of AgNPs alleviated the levels of catalase (CAT), ascorbate peroxidase (APX) and glutathione reductase (GR), along with enhancing the growth of plants. Besides, decreased H_2_O_2_, lipid peroxidation and ROS were observed in treated plants [96]. Consistent with these findings, in bananas a significant increase in concentrations of SOD, POD and CAT was observed in seedlings treated with AgNPs, as well as an increased content of chlorophyll and carotenoids. Higher concentrations of AgNPs also resulted in increased H_2_O_2_ and proline content [97]. Likewise, increase in plant height and seed germination have been suggested in summer savory [98]. Furthermore, enhanced sugar synthesis in tomato was reported by treatment of silver nanoparticles [99].

### 3.2. Nanoparticles in Abiotic and Biotic Stresses

Nanoparticles have been suggested as promising for alleviating the damage caused by abiotic and abiotic stress. In recent studies, metallic nanoparticles have shown many applications in plants. Enhanced plant growth and induced plant resistance against biotic stress by silica nanoparticles have been reported. Similarly, the use of copper, zinc oxide, and selenium nanoparticles as nano-fertilizers yielded excellent results [100,101,102,103]. Moreover, chitosan nanoparticles releasing nitic oxide have been demonstrated to be promising against salinity stress in maize plants [104]. Consistent with this, another study on soybean reported the improvement of plant growth under copper stress mediated by release of nitric oxide by chitosan nanoparticles [105]. Similarly, considering the biocompatibility and antimicrobial properties of silver and copper, their nanoparticles have been widely used in the amelioration of various biotic stressors [106,107,108,109,110,111,112,113,114,115,116,117,118,119,120,121,122,123,124,125].

Recent attempts have suggested the role of nanoparticles in promoting stress tolerance by acting as antioxidants or boosting the antioxidative system [126] (Figure 3). Various recent studies have confirmed the use of nanoparticles in alleviating ROS-induced stress by boosting the antioxidant system. These include AuNPs in wheat, ZnO NPs in peas, tomato, and okra, CeO_2_ and CuO NP in maize, corn, and soybean, Ca_3_(PO_4_)_2_ in beans, AgNP in pearl millet, biochar NP in wheat, Zeolite NPs in potato, chitosan NP in bitter melon, graphene oxide NPs in wheat and SiO_2_ NPs in peas [127,128,129,130,131]. The various nanoparticles and their antioxidant roles have been highlighted in the tables below (Table 1 and Table 2). These research projects have concluded that low concentration of NPs triggers detoxification of the ROS and activates antioxidant enzymes by upregulating the signaling genes [132].

**Table 1:** Different nanoparticles used in amelioration of varietal abiotic stresses in different plant species.

Abiotic Stress	Nanoparticle	Crop	Impact	References
Salt	SiO_2_	Tomato	Improved phenolics, chlorophyll and PAL activity	[127]
Drought	Fe_2_O_3_	Linseed	Decreased levels of H_2_O_2_ and MDA; enhanced activity of SOD, POD, CAT	[133]
Salt	Fe_3_O_4_	Drumstick tree	Decreased MDA, H_2_O_2_, lipid peroxidation	[134]
Salt	K_2_SO_4_	Alfalfa	Decreased electrolyte leakage, Improved antioxidant activity, increased proline	[135]
Drought	ZnO	Rice	Decreased MDA, lipid peroxidation	[136]
Salt	ZnO	Okra	Decreased accumulation of proline, enhanced photosynthetic pigments, improved activity of CAT and SOD	[137]
Heavy metal (Pb)	Si	Coriander	Decreased MDA, improved plant biomass	[138]
Drought	TiO_2_	Linseed	Increased carotenoids, chlorophyll; decreased lipid peroxidation, MDA and H_2_O_2_	[139]
Salt	Ag	Pearl millet	Increased proline and relative water content	[140]
Cold	TiO_2_	Chickpea	Decreased electrolyte leakage index	[141]
Flood	Al_2_O_3_	Soybean	Increased expression of proteins involved in lipid metabolism, protein degradation/synthesis and glycolysis	[142]
Salt	Si	Tomato	Alleviation of oxidative stress by upregulation of *P5CS*, *AREB*, *MAPK* and *CRK1*	[143]
Heat	Ag	Wheat	Decreased ROS	[144]
Drought	Cu	Maize	decreased ROS accumulation, increased total seed number	[145]
Heavy metal (Cd and Pb)	Fe_3_O_4_	Wheat	Increased activity of SOD and POD	[146]
Heavy metal (As)	Ti	Moong bean	Induced expression of CAT and SOD, upregulation of antioxidant related genes	[147]
Heavy metal (As)	ZnO	Soybean	Increased activity of APX, GR, CAT and SOD	[148]
Heavy metal (Cd)	TiO_2_	Maize	Decreased Cd accumulation along with increased activity of antioxidant system	[149]
Heavy metal (Cr)	ZnO	Wheat	Increased activity of APX, CAT, POD and SOD	[150]
Heavy metal (As)	Fe	Rice	Improved defense enzymes and glyoxalase machinery	[151]
Heavy metal (Cd)	ZnO	Wheat	Reduced electrolyte leakage, enhanced activity of SOD and POD	[152]
Drought	ZnO	Safflower	Increased grain yield biomass yield and number of seeds	[153]
Salinity	Si NPs	Tomato	Increased content of photosynthetic pigments; Higher biomass and yield	[154]
Drought	ZnO	Wheat	Foliar application at 100 and 150 ppm resulted most effective management of drought stress	[155]
Salinity	GO-Pro NPs	Grapes	Foliar application at 100 mM reduced electrolyte leakage, proline and upregulated AOE,	[156]
Heat and Drought	Se NPs	Wheat	Foliar application at 10 mgL^−1^ improved GE, TR and photosynthetic machinery	[157]
Heavy metal (Cd)	Si NPs	Wheat	Improved photosynthetic pigments and AOEs	[158]
Drought	ZnO and SiO_2_	Potato	Foliar application of ZnO at 100 mg L^−1^ increased productivity and enhanced quality	[159]
Heavy metals (Cd, Pb)	Zn, Se, Si	Sage	Improved plant weight, RWC, EL and EO	[160]
Salinity	Si NPs and MT	Cauliflower	Improved chlorophyll content and osmolyte levels	[161]
PEG induced Drought stress	Kn-ZnO NPs	Mung bean	Upregulation of osmolyte levels and antioxidant system	[162]
Salinity	Si NPs	Lemon grass	Amplification of SC and photosynthetic CO_2_ assimilation	[163]
Heat	ZnO	Rice	Decreased ABA levels, improved tolerance to osmotic stress	[164]
Drought	Si NPs	Wheat	Upregulation of defense related genes *DREB2*, *MYB33*, *MYB3R*, *WRKY 19*, *SnRK2.4*	[165]
Drought	NNS	Tomato	Foliar application at 1%, 3% and 5% gradually increased AOE activity	[166]
Salinity	Ag NPs	Pearl millet	Upregulation of SOD, CAT and POD	[167]
Heavy metal contaminated Wastewater	Se	Carrot	Decreased free proline, MDA, hydrogen peroxide and increased soluble protein, β-carotene	[168]
Drought	Si, Zn, Zeolite	Coriander	Improved photosystem II, water used efficiency, leaf chlorophyll and transpiration rate	[169]

The table summarizes various nanoparticles used in mitigation of abiotic stress in different crops. Abbreviations: Ag—silver; Al_2_O_3_—aluminum oxide; AOE: anti-oxidant enzymes: AREB—ABA response element binding protein; As—arsenic; CAT—catalase; Cd—cadmium; CRK1—cysteine rich receptor-like protein kinase 42; Cr—chromium; Cu—copper; EL—electrolyte leakage; EO: essential oil; Fe_2_O_3_ and Fe_3_O_4_—iron oxide; GE—gas exchange; GO-Pro NPs—proline functionalized graphene oxide nanoparticles; GR—glutathione reductase; H_2_O_2_—hydrogen peroxide; K_2_SO_4_—potassium sulfate; Kn-ZnO NPs—kinetin capped zinc oxide nanoparticles; MAPK—mitogen-activated protein kinase; MDA—malondialdehyde; MT—melatonin; NNS—nano-nutrient solution; PAL—phenylalanine ammonia lyase; P5CS—pyrroline-5-carboxylate synthetase 1; Pb—Lead; POD—peroxidase; RWC—relative water content; SC—stomatal conductance; Se—selenium; SOD—superoxidase dismutase; Si—silicon/silica; SiO_2_—silicon dioxide; TiO_2_—titanium dioxide; TR—transpiration rate; ZnO—zinc oxide.

**Table 2:** Various nanoparticles used in mitigating biotic stress in different plants.

Biotic Stress	Nanoparticle	Crop	Impact	References
*Magnporthe oryzae*	ZnO	Rice	Inhibition of appressorium formation, upregulation of *OsNAC4*, *OsPRO10*, *OsKSL4*, *OsPR1b* genes involved in resistance	[164]
*Bipolaris sarokiniana*	Se	Wheat	Increased chlorophyll content, membrane stability index, leaf surface area, root length	[170]
*Xanthomonas oryzae*	Ag	Rice	decreased effects of ROS by boosting cellular antioxidative system	[171]
*Rhyzopertha dominica* and *Sitophilus granarius*	CuO	Wheat	Increased concentration of leaf pigments, Increased activity of antioxidant enzymes viz SOD, APX, POD; increased insect mortality	[172]
*Puccinia striiformis*	TiO_2_	Wheat	Downregulation of proteins involved in production of ROS	[173]
*Fusarium oxysporum* and *Aspergillus niger*	Si	Maize	Increased phenolics, POD and PPO	[174]
*Fusarium fujikuroi*	Si	Rice	Improved electrolyte leakage and POD activity	[175]
*Fusarium oxysporum*	ZnO	Chickpea	Increased antioxidant activity and activation of SOD, POD, CAT	[176]
*Phytophthora nicotianae* and *Thielaviopsis basicola*	MgO	Tobacco	Induced ROS production	[177]
*Meloidogyne incognita, Pectobacterium betavasculorum,* and *Rhizoctonia solani*	SiO_2_	Beetroot	Enhanced chlorophyll content and improved activity of defense related enzymes	[178]
*Rhizoctonia solani*	Ca_3_(PO_4_)_2_, SiO_2_ and CuO	Potato	Boosted activities of POD, PPO, CAT and chitinase enzymes	[179]
*Fusarium andiyazi*	Chitosan	Tomato	Upregulation of PR genes, activation of SOD and related antioxidant genes	[180]
*Alternaria solani*	AgNP	Tomato	Increased activity of antioxidant enzymes SOD, CAT, APX, PAL, POD, PPO	[181]

The table summarizes various nanoparticles used in the mitigation of various biotic stress in different crops. Abbreviations: Ag—silver; APX—ascorbate peroxidase; Ca_3_(PO_4_)_2_—calcium phosphate; CAT—catalase; CuO—copper oxide; MgO—magnesium oxide; PAL—phenylalanine ammonia lyase; POD—peroxidase; PPO—polyphenol oxidase; PR genes—pathogenesis related genes; ROS—reactive oxygen species; SOD—superoxidase dismutase; Se—selenium; Si—silicon/silica; SiO_2_—silicon dioxide; TiO_2_—titanium dioxide; ZnO—zinc oxide.

## 4. Nano-Assisted Agricultural Practices

### 4.1. Nano-Delivery

**Figure 4:**
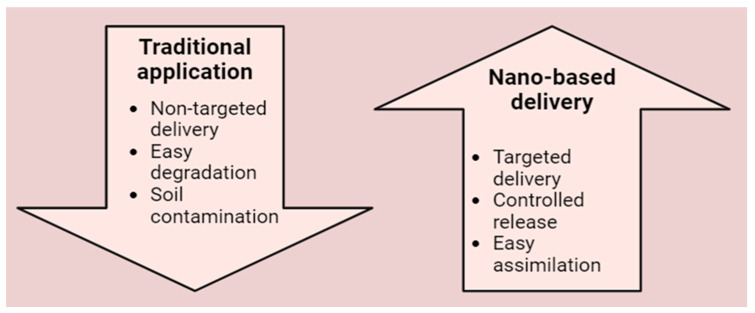
Advantages of nano-based delivery of fertilizers as compared to traditional methods.

Recent attempts have been made to design nano-structured carriers, utilizing materials like nano-clays and polymeric nanoparticles for controlled-release formulations of fertilizers and pesticides [182]. These nano-carriers protect active ingredients, enabling gradual release and targeted delivery to plants, reducing environmental impact, and optimizing resource utilization [183]. Innovations include the use of controlled release nano-fertilizers (CRFs) [183]. CRFs can deliver nutrient to plants for extended days to months, besides protection from the release of fertilizers in environment, contributing to their applicability in sustainable agricultural practices (Figure 4) [184,185]. Various nanomaterials, including quantum dots, graphene, and carbon-nanotube, due to their small size and unique properties, have been adversely used in controlled release applications [186,187]. Moreover, the nano-encapsulation technique has been recently used to protect seeds from pathogens, enhance nutrient uptake during germination, and to provide improved drought tolerance [188,189,190,191]. Nano-fertilizers possessing phosphorous, potassium and nitrogen have been reported to improve growth and productivity in plants [192]. Likewise, nano-fertilizers have been suggested to improve tolerance from biotic and abiotic stresses in plants [6]. Advances have also focused on using nanomaterials for seed delivery and improved seed germination [193]. Nano-encapsulation techniques employ materials like lipid-based nanoparticles and biodegradable polymers to protect seeds [194]. Recent studies have explored the potential of nanomaterials, such as zinc oxide nanoparticles, in seed priming to enhance early growth and stress tolerance in crops [195,196]. A study on bitter almond seedlings reported the successful germination of seeds treated with nano-urea modified hydroxyapatite nanoparticles under salinity stress [197]. Similarly, for the growth of corn seedlings, a copper oxide-based tenorite nano-fertilizer demonstrated effective results [198]. Moreover, for improvement of biomass in maize, a chitosan based sustained release nano-fertilizer was also developed [199]. The use of zerovalent iron nano-fertilizer in aromatic rice improved germination [200].

### 4.2. Nano-Monitoring

**Figure 5:**
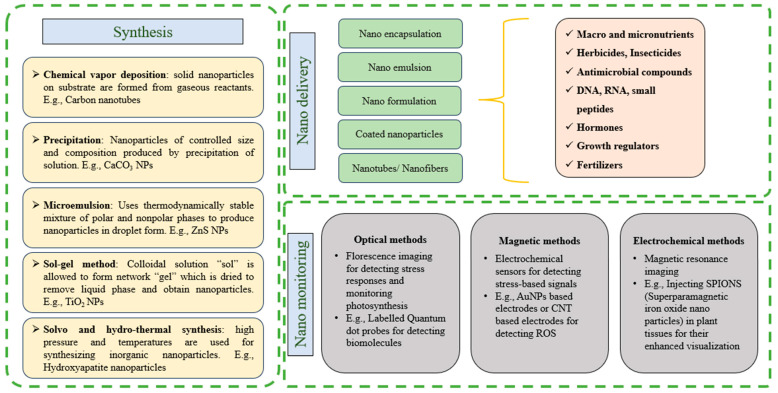
Image describing diverse applications of nanoscience in agriculture highlights nanoparticle synthesis methods and their deployment in monitoring crop plants with enhanced techniques.

For maintaining sustainability in agriculture, development of new techniques is imperative. These advancements have led to the development of nano-based biosensors “nano-biosensors”, which have the potential to sense their environments [201]. In agriculture, nano-based sensors have evolved to provide real-time monitoring of crucial parameters. The use of these nano-biosensors, due to their ability to sense, process and detect changes, has contributed to the growth of “smart agriculture” and “precise farming” [202,203]. Carbon nanotube-based sensors are gaining attention for their applications in soil sensing, offering high sensitivity and selectivity for detecting nutrient levels and moisture content [204,205,206]. Similarly, quantum dot nano-biosensors have been used to detect mycotoxins in barley and corn. A surface plasmon resonance biosensor has been used to detect the *Cymbidium* Mosaic virus [207]. Molecular imprinted polymer-based nano-biosensors have been employed to sense polyphenols in vegetables [208]. Graphene-based molecular imprinted polymer nano-biosensors have been used to detect chlorothalonil and chlorpyrifos methyl pesticides [209]. Likewise, an acetylcholine esterase biosensor has been used to detect parathion, parazoan and methyl-parathion pesticides. Moreover, a nano-biosensor “artificial nose” has been reported to sense released organic compounds and detect pathogens based on those compounds [210]. Consistent with these studies, current attempts are ongoing on for the integration of nanoscale sensors with wireless communication systems, enabling remote and continuous monitoring of agricultural fields. Furthermore, nano-farming strategies have aimed at a holistic integration of nanotechnology into farming practices [211]. These include the use of engineered nanomaterials, such as functionalized nanoparticles and nano-composites, for enhancing soil fertility, water retention, and nutrient availability [212] (Figure 5). The ongoing research is investigating the potential of nanoscale delivery systems not only for nutrients and pesticides but also for beneficial microorganisms, promoting sustainable and eco-friendly farming practices.

## 5. Current Challenges and Limitations

The unique properties of nanoparticles contribute to their applicability in sustainable agricultural practices. The ultra-small particle size of NPs makes them immensely useful for deterioration of ROS at enzymatic, non-enzymatic, biochemical, and molecular levels. However, contrastingly, the same properties of NPs adversely affect the health of plants, as well as humans [213,214,215]. These challenges and limitations hinder their widespread use and acceptance. One major concern is the potential ecotoxicity of nanoparticles, which poses a threat to the environment [216]. The impact on soil microorganisms, aquatic ecosystems, and non-target organisms questions the overall safety and sustainability of nanoparticle applications. Addressing these concerns necessitates a thorough assessment of the ecological consequences associated with nanoparticle exposure. Moreover, the absence of standardized protocols for assessing nanoparticle toxicity complicates the regulatory approval process [217]. A still imperative challenge is the establishment of clear and universally accepted guidelines for nanoparticle safety testing. The lack of such regulatory frameworks hinders the industrial applicability of nanoparticles [218,219]. Understanding the fate and transport of nanoparticles in the environment is another critical challenge. The long-term impacts of nanoparticles on ecosystems remain uncertain without comprehensive knowledge of their fate and transport dynamics. This knowledge gap makes it challenging to predict and mitigate potential associated adverse effects. Cost and scalability issues further contribute to the limited adoption of nanoparticles on a large scale. Some nanoparticles are highly expensive to produce, restricting their practical applicability. For overcoming these challenges, the development of cost-effective and scalable synthesis methods, aligning with safety for implementation, are necessary. Additionally, the variable responses of different plant species to different nanoparticle exposure makes it more difficult to assess environmental impact [220,221,222]. An extensive understanding of these species-specific responses is crucial for predicting and managing the potential consequences accurately. Henceforth, these hurdles need to be addressed for ensuring the responsible, safe and sustainable use of nanoparticles in agriculture.

## 6. Future Directions

Future advancements in nanoparticles and plant interactions should prioritize long-term ecotoxicity studies, standardized testing protocols for regulatory approval, and the development of advanced tracking techniques to monitor nanoparticle fate and transport in real-time. Additionally, there is an urge for innovative and cost-effective synthesis methods, precision agriculture approaches that consider species-specific responses, and a deeper understanding of the nano–bio interface. Exploring nano-enabled nutrient delivery systems for plants and developing integrated risk assessment models are crucial for sustainable agriculture practices. Intense exploration of the benefits and risks of nanoparticles, along with interdisciplinary collaborations, will play a pivotal role in expanding nanoparticle applications.

## 7. Conclusions

Nanoparticles, based on their size, composition, and sensitivity, interact with plants in a number of ways. These interactions result in various anatomical, morpho-physiological and biochemical changes that are directly related to the overall efficiency of crop plants. Some of the beneficial effects of nanoparticles in plants include enhanced growth, increased fresh biomass, improved chlorophyll content, improved metabolism, increased antioxidant potential upregulation and improved expression of stress-related genes, which are crucial for stress resilience by alleviating protein and chlorophyll and promoting nitrogen metabolism. Despite the effectiveness of nanoparticles in ameliorating various stresses, most of these studies are still in the laboratory stage. The increased applicability of nanoparticles is of concern due to their unexpected effects on the environment, as well as their accumulation in edible plant organs, which pose serious risks of bioaccumulation in the food chain. Hence, more efforts to develop proper evaluation methodologies for evaluating the effects and predicting the fate of nanoparticles is required. Additionally, the standardization of the acceptable limits of nanoparticles in their wide range of applicable areas is needed. Future studies should focus on the development of non-toxic, ecologically safe, affordable, stable, and self-degradable nanoparticles for commercializing nanotechnology from laboratories to the agricultural fields. A multidisciplinary and collaborative approach involving researchers, policymakers, and industry stakeholders is essential to navigate these complexities and unlock the full potential of nanoparticles while ensuring environmental sustainability and safety.

## Data Availability

Not applicable.
